# Development and validation of an integrated machine learning model for recurrence-free survival prediction in clear cell renal cell carcinoma

**DOI:** 10.1016/j.tranon.2026.102937

**Published:** 2026-07-29

**Authors:** Yun-Lian Wang, Feng Liu, Qian-Biao Gu, Qiang Liu, Ying Yang, Fang Cheng

**Affiliations:** a VIP Department, Affiliated Tumor Hospital of Xinjiang Medical University, Urumqi, Xinjiang, 830011, China; b Department of Urology, The Central Hospital of Shaoyang, Shaoyang, Hunan, 422000, China; c Imaging Center, Hunan Provincial People's Hospital, First Affiliated Hospital of Hunan Normal University, Changsha, Hunan, 410005, China; d Urology Center, First Affiliated Hospital of Xinjiang Medical University, Urumqi, Xinjiang, 830054, China; e Tumor Center, People's Hospital of Xinjiang Uygur Autonomous Region, Urumqi, Xinjiang, 830001, China

**Keywords:** Clear cell renal cell carcinoma, Machine learning, Radiomics, Deep learning, Recurrence-free survival, Prediction model

## Abstract

Background: Integrated multimodal systems improve recurrence-free survival (RFS) prediction in surgically resected clear cell renal cell carcinoma (ccRCC) to overcome traditional model limitations. Methods: A total of 1284 patients were enrolled and divided into training (n = 738), internal validation (n = 317), and external test (n = 229). A machine learning (ML)-based model combined clinical data, radiomics signature (Rad-sign), and deep learning signature (DL-sign) to predict recurrence at 3-, 5-, and 7-year post-surgery. Radiomics features were extracted from CT images using ML, with the Rad-sign showing the best performance. A three-dimensional vision transformer (3D-ViT) for volumetric feature extraction outperformed ResNet models. Results: Cox regression identified clinical features associated with RFS (*p* < 0.05). An optimal clinical-DL nomogram was chosen from 8 ML algorithms via 5-fold cross-validation. Clinical features alone had limited predictive power (external test AUC: 0.576, 95% CI: 0.472–0.672), while radiomics modeled by Naive Bayes (NB) performed better (AUC: 0.728). Rad-sign's C-index was 0.729, and the 3D-ViT model achieved an external test AUC of 0.846. The nomogram's AUCs for predicting 3, 5, and 7-year RFS post-surgery were 0.920, 0.935, and 0.938, with C-indices of 0.890 and 0.910, respectively. It effectively stratified risk groups, outperforming UISS and SSIGN via calibration and decision curve analysis. Conclusion: The ML-based clinical-DL model shows promise for accurate ccRCC RFS prediction, which may aid in personalized risk assessment and decision-making.

## Highlights

• Integrated ML framework fuses clinical features and DL-sign for ccRCC RFS prediction.
• 3D-ViT outperforms ResNet variants, delivering high external test AUC of 0.846 for DL signature.
• Combined nomogram achieves excellent external C-index of 0.910 with robust multi-year RFS prediction.
• Model outperforms UISS/SSIGN, enabling precise ccRCC risk stratification and clinical decision support.

## Introduction

Clear cell renal cell carcinoma (ccRCC) develops in the renal tubules, and is a highly aggressive subtype of renal cell carcinoma (RCC), originating in the renal tubules [1]. Partial or radical nephrectomies are highly curative for RCC; however, disease recurrence, often manifesting years after surgery, affects 20–40% of patients and significantly impacts long-term survival and quality of life [2,3]. Consequently, identifying high-risk patients for recurrence accurately continues to be a crucial but challenging component of patient survival, postoperative monitoring strategy optimization, and adjuvant therapy selection.

Traditional prognostic models for ccRCC primarily rely on clinicopathological features such as tumor stage, nuclear grade, and tumor size. The UCLA Integrated Staging System (UISS) and the Stage, Size, Grade, and Necrosis (SSIGN) score are established prognostic models widely used for ccRCC prognosis, and use conventional clinical and pathological parameters to predict patient outcomes [4,5]. Based on research, they offer readily accessible indicators, simple calculation protocols, and excellent external generalizability, thus serving as benchmarks for the development and validation of novel prognostic models [6,7]. The SSIGN score is a model developed to predict cancer-specific survival for ccRCC patients. It is further utilized in clinical guidelines for risk stratification and to evaluate the verification of biomarkers in clinical trials [8]. However, these models are limited in predictive accuracy, as evidenced by reported C-indices of around 0.82 in external validation studies [9]; thus highlighting the need for more comprehensive prediction tools that capture the biological heterogeneity driving recurrence risk.

Radiomics is a specialized branch of medical imaging that extracts high-dimensional quantitative features from standard non-invasive medical scans to aid in cancer diagnosis, prognosis, and treatment monitoring. By extracting complex tumor characteristics such as tumor texture, shape, and intensity patterns, it captures tumor heterogeneity and microenvironment changes, offering insights into tumor biology that are often invisible. This improves clinical observation and provides objective biomarkers that complement traditional clinical parameters [10,11]. Recent studies have demonstrated the potential of radiomics to predict treatment response, survival outcomes, and disease recurrence across various cancer types, including RCC [12,13]. Advanced radiomics features, including fractal dimensions and curvature-derived topological characteristics, provide additional insights into tumor complexity and spatial organization.

Deep learning (DL) has transformed medical image analysis by automating the extraction of hierarchical, multi-scale features from raw imaging data, allowing for improved accuracy in classification and detection. Vision Transformers (ViTs), initially developed for natural image processing, have been adapted for medical imaging tasks and have demonstrated superior performance compared with traditional convolutional neural networks [14,15]. Three-dimensional ViTs (3D-ViTs) improve CT analysis by processing full 3D volumetric data, overcoming two-dimensional (2D) limitations by capturing spatial relationships across all dimensions. The self-attention mechanism in transformers allows the 3D-ViT-based model to focus on relevant anatomical structures and pathological features, potentially providing complementary information for hand-crafted radiomics (HCR) approaches [16].

Integrating clinical data with HCR features [17] and the incorporation of deep learning signatures (DL-sign) [18] signify a transformative shift towards multi-modal machine learning (ML) in the field of precision oncology. Although individual studies have investigated single-modality approaches, comprehensive comparisons of various ML algorithms across multiple feature types with rigorous multi-center validation are limited in ccRCC. Additionally, most existing models have not undergone head-to-head validation against established prognostic scores.

This study systematically develops and validates an integrated ML framework for recurrence-free survival (RFS) prediction in ccRCC patients. Comprehensive radiomics features, including texture, shape, fractal dimensions, and topological properties, were extracted. DL-sign was generated from multiple architectures, including 3D-ViT and ResNet variants. Fourteen ML algorithms were evaluated, and their performance was compared against established prognostic models (UISS and SSIGN) using a multicenter dataset with independent external validation

## Methods

### Patient population and study design

We retrospectively analyzed the data of patients who underwent partial nephrectomy or radical nephrectomy for histopathologically diagnosed ccRCC. The patients were recruited from 5 hospitals and enrolled in this multicenter retrospective study. Inclusion criteria were as follows: (1) patients pathologically diagnosed ccRCC; (2) available preoperative contrast-enhanced CT imaging; (3) complete clinicopathological data; (4) adequate follow-up duration. A total of 1284 eligible patients were included in the final analysis, with 581 patients receiving partial nephrectomy and 703 patients receiving radical nephrectomy. Surgical approach selection was guided by the *Chinese Guidelines for the Diagnosis and Treatment of Urological and Andrological Diseases (RCC Edition)*, using the validated R.E.N.A.L. nephrometry score. This system stratifies tumor complexity by assessing size, growth pattern, proximity to the collecting system, and anatomical location, thereby facilitating consistent and clinically appropriate decisions regarding nephron-sparing versus radical surgery.

Patients from four centers (n = 1055) were combined and randomly divided into the training cohort (n = 738, 70%) and internal validation cohort (n = 317, 30%). The group of patients from Hunan Provincial People's Hospital was used as an independent external test cohort (n = 229) to evaluate the generalizability of the model. This study was approved by the Ethics Committee of the Affiliated Tumor Hospital of Xinjiang Medical University in accordance with the Declaration of Helsinki. Centralized ethical approval was obtained for all five participating hospitals together with formal data-sharing agreements to ensure all institutions adhered to and complied with medical privacy and security protocols for this study. The requirement for written informed consent was waived, as all relevant patient data were kept anonymous, as well as due to the retrospective nature of this multicenter study.

### Clinical data collection

Clinical and pathological variables were obtained from medical records, including patient demographics (age, gender), tumor characteristics (pathologic tumor size, nuclear grade, presence of hemorrhage, necrosis, nerve invasion, and lymphovascular invasion), and Tumor-Node-Metastasis (TNM) staging, which was classified based on the 8th edition (2017) of the American Joint Committee on Cancer (AJCC) TNM staging system for RCC [19]. Specifically, nodal status was pathologically confirmed postoperatively, with N1 disease defined as the presence of lymph node metastasis in postoperative specimens and N0 disease as no lymph node metastasis. Lymph node dissection was performed in patients with suspected regional lymph node metastasis, confirmed by preoperative enhanced CT/MRI or intraoperative exploration. Laboratory parameters, including neutrophil count, monocyte count, lymphocyte count, platelet count, hemoglobin, lactate dehydrogenase, albumin, serum calcium, and fibrinogen, were also collected. UISS and SSIGN scores were calculated for all patients based on established algorithms [4,5].

All patients received standardized and regular postoperative follow-up. The follow-up protocol was formulated in accordance with routine clinical guidelines for RCC. Systemic examinations were conducted every 3 months within 1–2 years postoperatively, every 6 months during 3–5 years postoperatively, and annually after 5 years postoperatively. Follow-up assessments included contrast-enhanced computed tomography (CT) of the chest, abdomen and pelvis, whole-body bone scan, and contrast-enhanced cranial magnetic resonance imaging to monitor tumor recurrence. Tumor recurrence was strictly judged based on the Response Evaluation Criteria in Solid Tumors version 1.1 (RECIST 1.1), which is widely applied in RCC. Recurrence or metastasis was confirmed when new lesions were detected in the surgical area, lung, bone, lymph nodes, intracranial region, or other organs via imaging examination, and these lesions were radiologically suspected as tumor recurrence or metastasis. The final diagnosis was comprehensively determined by clinicians combining imaging findings, clinical symptoms, and tumor marker levels.

### Image acquisition and preprocessing

CT images were acquired using various CT scanners across the participating centers from multiple medical centers. Only the corticomedullary phase (CMP, also known as the arterial phase) images of contrast-enhanced CT were selected for tumor segmentation and radiomic feature extraction, as this phase is widely adopted in numerous previous RCC-related radiomics and DL studies [20,21] to ensure reliable feature stability and favorable predictive model performance. Tumor ROIs were manually delineated by two experienced radiologists following unified imaging criteria, with prior standardized training on the segmentation workflow to ensure consistency. All segmented images were resampled to a uniform voxel size of 1 mm^3^ to ensure consistent volumetric resolution across different scanners and imaging protocols. This standardization reduces the difficulty of model generalization across multi-center data. To further mitigate potential batch effects and inter-scanner variability inherent to multi-center CT imaging data, we performed additional image-level preprocessing for all CT scans, including unified soft-tissue window grayscale normalization and Z-score standardization. This pipeline eliminated systematic discrepancies in reconstruction parameters, contrast settings, and pixel intensity distribution biases stemming from heterogeneous imaging devices and scanning protocols. Inter-observer variability in segmentation was quantitatively assessed, and all inconsistent segmented regions were resolved through collective consensus discussion. At the feature level, the ComBat algorithm was adopted to uniformly harmonize extracted radiomic features after feature extraction and prior to model training. Such systematic post-processing and harmonization strategies effectively controlled confounding factors attributed to inter-center equipment differences and inconsistent scanning procedures, thereby substantially eliminating residual multi-center heterogeneity and batch effects. This rigorous preprocessing workflow ensures the robustness and generalizability of the subsequent predictive models. Data augmentation techniques were employed during model training, including random cropping, rotation (±10°), and flipping, to enhance model robustness and minimize the risk of overfitting.

### Radiomics feature extraction and selection

The radiomics feature extraction in this study included HCR feature extraction. HCR features were extracted using Python 3.8 and the open-source Pyradiomics package (http://pypi.org/project/pyradiomics/), and the textural features extracted included various types of matrix features such as first-order features, shape, Gray-Level Co-occurrence Matrix (GLCM), Gray-Level Size Zone Matrix (GLSZM), Gray-level Run Length Matrix (GLRLM), Neighboring Gray Tone Difference Matrix (NGTDM), and Gray-Level Dependence Matrix (GLDM). Detailed feature descriptions are available in the Pyradiomics documentation (http://pyradiomics.readthedocs.io). Fractal dimension features and curvature-based topological features were also extracted for subsequent model construction.

To select features with rich information, the independent *t*-test was used to select features with good statistical significance *p* > 0.05, next, Pearson correlation analysis was performed to remove highly redundant features (r > 0.9). Then LASSO logistic regression algorithm with penalty parameter tuning conducted by 10-fold cross-validation was applied to select the most useful predictive features with non-zero coefficients.

After feature fusion and screening, multiple ML classification models were established based on Python 3.8 and PixelMedAI platform, including Logistic Regression (LR), Support Vector Machine (SVM), K Nearest Neighbor (KNN), Random Forest (RF), Extremely Randomized Trees (ExtraTree), eXtreme Gradient Boosting (XGBoost), Light Gradient Boosting Machine (LightGBM), and Multi-Layer Perceptron (MLP). 5-fold cross-validation was adopted to optimize hyperparameters and reduce overfitting risk. The probability output of the optimal model was defined as the Rad-sign.

### DL architecture

The architecture of the traditional ViT was adapted and optimized for this study. Unlike conventional 2D-ViT image processing models, the adapted model fully incorporates the 3D spatial characteristics of the image, effectively utilizing voxel information across all spatial dimensions. During network input, the 3D image partitioned into fixed-size 3D patches to split the whole volumetric image into small overlapping cubic units. These 3D patches are then converted into one-dimensional (1D) vectors through a flattening operation, and the local structural information of each patch is encoded into a high-dimensional embedding representation. Positional encoding information is added after patches are embedded using a linear layer to preserve the original spatial positional information within the embedding representation. This ensures that the model maintains the spatial context that is essential for medical picture interpretation.

The network integrates a learnable class token (cls token) that serves as a surrogate for the global information regarding the overall features of the input 3D image. This cls token is introduced at the initiation of the sequence of all patch embeddings, followed by positional encoding of the entire sequence to accurately reflect the ordinal information of each patch in 3D space. Building on this, a Dropout layer was employed to implement regularization, thereby mitigating the risk of overfitting during model training and enhancing the generalizability of the model to unseen data.

The core component of the model is the Transformer encoder, which processes the sequence of patch embeddings to capture complex global image features, a critical capability for identifying subtle pathological patterns in medical images. The Transformer is composed of several stacked encoder layers, each comprising a multi-head self-attention (MHSA) mechanism and a feedforward network (FFN). The self-attention mechanism captures global feature interactions in CT images by calculating the correlation weights between individual patch embeddings. Specifically, the MHSA module first applies LayerNorm normalization to the input sequence to stabilize training, then employs the Softmax function to compute the attention distribution, while preserving input information through residual connections to prevent gradient vanishing. In addition, the FFN consists of multiple linear transformation layers, GELU nonlinear activation functions and Dropout operations to strengthen feature representation capability. With residual connections and layer-wise regularization, the full Transformer encoder maintains gradient stability during training and improves model robustness.

In classification tasks, the model adopts two feature aggregation strategies. The first directly employs the class token embedding as the final feature, while the second aggregates all patch embeddings via mean pooling. Under both strategies, the aggregated features are fed into a lightweight decoder composed of a LayerNorm layer and a fully connected layer to obtain final category predictions.

We compared ViT with mainstream classical models including ResNet-18, ResNet50, and ResNet101. The probability output from the optimal model is defined as the DL-sign. To improve clinical interpretability, we adopted Shapley analysis to generate saliency maps, which intuitively visualize the model's attention regions targeting tumor lesions.

All DL model training and inference in this study were implemented on a server with an NVIDIA GeForce RTX 5060 Ti GPU (16 GB memory) and Ubuntu 20.04 system, using PyTorch 2.1 as the core DL framework. The Vision Transformer model was trained for 200 epochs with a training duration of 6–8 h, and the single-image inference time was 50–80 ms, which satisfies the real-time operation requirements of clinical decision support scenarios.

### ML model development

Clinical features significantly associated with postoperative recurrence were identified through univariate and multivariate Cox regression analyses (*p* < 0.05). Subsequently, multiple ML classification models were developed using the PixelMedAI platform on Python 3.8. The evaluated algorithms included:

• Logistic Regression (LR)
• Support Vector Machine (SVM) with multiple kernels (linear, polynomial, radial basis function, sigmoid)
• K-Nearest Neighbors (KNN)
• Naive Bayes (NB)
• Decision Tree (DT)
• Random Forest (RF)
• Extremely Randomized Trees (ExtraTree)
• eXtreme Gradient Boosting (XGBoost)
• Light Gradient Boosting Machine (LightGBM)
• Multi-Layer Perceptron (MLP)

To minimize the risk of overfitting, 5-fold cross-validation was used to select optimal hyperparameters for each algorithm. Models were trained separately using clinical features and radiomics features, with fourteen ML algorithms evaluated for each feature set. A combined prediction model incorporating clinical features and DL-sign was established and visualized as a nomogram.

Different from DL models, all traditional ML models in this study adopted CPU-based training and inference, with low computational resource consumption. For future application and promotion, the established models will be packaged into standardized interfaces and deployed on the hospital's private cloud server to support multi-terminal access and multi-center clinical application.

### Integrated Cox regression model and nomogram development

An integrated Cox proportional hazards regression model was constructed to predict RFS in ccRCC. First, univariate Cox regression analysis was performed on all clinicopathological variables to screen for prognostic factors associated with postoperative recurrence, with a statistical significance threshold set at *p* < 0.05. All significant variables identified in the univariate analysis were subsequently included in multivariate Cox regression analysis to determine independent prognostic factors. The final integrated model incorporated independent clinical features (age, pathological tumor size, nuclear grade, necrosis, and T stage) and the DL-sign derived from the optimal ViT model. Model construction was conducted in the training cohort (n = 738) using the Breslow method to handle tied events, and the model was validated in both the internal validation cohort (n = 317) and external test cohort (n = 229). Based on the final Cox regression model, a quantitative nomogram was established to achieve individualized prediction of 3-, 5-, and 7-year RFS probabilities for patients. The discriminative ability of the model was evaluated using Harrell's concordance index (C-index), and calibration curves were plotted to verify the consistency between predicted and actual RFS outcomes. The optimal cut-off value of the prognostic signature was determined by maximizing log-rank test statistics, which was applied to stratify patients into clinically distinct risk subgroups for survival analysis.

### Statistical analysis

All statistical analyses in this study were performed using R software (version 4.0.2; https://www.r-project.org) with the survival and rms packages, and Python software (version 3.8; https://www.python.org). For baseline data description, continuous variables were presented as mean ± standard deviation (SD), while categorical variables were expressed as count (n) and percentage (%). Specifically, Student's *t*-test was adopted for analyzing normally distributed quantitative data, and Levene's test was used to evaluate variance homogeneity; non-normally distributed quantitative data were analyzed via the Mann-Whitney U test, and the chi-square test was used for inter-group comparison of categorical data. A two-sided *p-value* <0.05 was defined as statistically significant for all analyses. Multiple validated methods were applied to comprehensively evaluate model performance and clinical value. Receiver operating characteristic (ROC) curves and the corresponding area under the curve (AUC) were used to assess the classification efficacy of the prediction model. Decision curve analysis (DCA) was performed to quantify the net clinical benefit of the model at different threshold probabilities, so as to evaluate its clinical utility. Harrell's C-index was utilized to assess the RFS prognostic discrimination ability of all models. The optimal cut-off values for DL-sign and Rad-sign in the training cohort were determined by traversing all potential cut points and adopting the log-rank test to screen for the threshold with the minimum *p*-value (maximal inter-group survival difference), and the unified cut-off values were applied for patient stratification in validation cohorts. Kaplan-Meier (KM) survival analysis with the Log-rank test was further used to evaluate the risk stratification performance of all prognostic models, including the Rad-score, DL-sign, established clinical scoring systems (UISS, SSIGN), and the integrated Cox nomogram, by exploring RFS differences between risk subgroups stratified according to optimal cut-off values. Calibration curve analysis was performed to assess the calibration degree between nomogram-predicted RFS probabilities and observed clinical outcomes. Time-dependent ROC analysis was conducted to evaluate the prognostic predictive ability at 3-, 5-, and 7-year RFS time points selected according to data distribution characteristics.

## Results

### Patient characteristics

The final cohort was composed of 1284 ccRCC patients, with 738 in the training cohort, 317 in the validation cohort, and 229 in the external test cohort. The mean age was 57.0 years (SD: 11.2), with 62.9% of the patients being male (n = 807). The median tumor size was 4.0 cm (IQR: 3.0–5.7 cm), with a range of 0.2–18.0 cm and a mean size of 4.56±2.49 cm. Nuclear grade distribution showed 14.9% grade 1 (n = 191), 61.4% grade 2 (n = 788), 20.8% grade 3 (n = 267), and 3.0% grade 4 (n = 38) tumors. The T stage distribution included 78.1% T1 (n = 1003), 5.9% T2 (n = 76), 13.2% T3 (n = 169), and 2.8% T4 (n = 36). The N stage distribution showed 93.4% N0 (n = 1199) and 6.6% N1 (n = 85). None of the patients had detectable distant metastasis (M0) (n = 1284, 100.0%). The median follow-up duration was 39.2 months (IQR: 20.3–62.8 months). The number of patients at risk for recurrence was 120 at 3 years, 77 at 5 years, and 43 at 7 years after treatment.

### Clinical feature analysis

Univariate Cox regression identified multiple clinical features significantly associated with RFS, including gender (HR: 1.334, 95% CI: 1.002–1.774, *p* = 0.048), age (HR: 1.028, 95% CI: 1.015–1.040, *p* < 0.001), pathologic tumor size (HR: 1.340, 95% CI: 1.295–1.387, *p* < 0.001), nuclear grade (HR: 2.199, 95% CI: 1.834–2.636, *p* < 0.001), necrosis (HR: 2.520, 95% CI: 1.762–3.605, *p* < 0.001), nerve invasion (HR: 7.550, 95% CI: 3.107–18.343, *p* < 0.001), lymphovascular invasion (HR: 2.456, 95% CI: 1.551–3.891, *p* < 0.001), T stage (HR: 1.804, 95% CI: 1.664–1.955, *p* < 0.001), and most laboratory parameters remained independently prognostic (Table 1). In multivariate analysis, age (HR: 1.014, 95% CI: 1.004–1.024, *p* = 0.006), pathologic tumor size (HR: 1.147, 95% CI: 1.098–1.199, *p* < 0.001), nuclear grade (HR: 1.313, 95% CI: 1.126–1.531, *p* = 0.001), nerve invasion (HR: 3.343, 95% CI: 1.308–8.548, *p* = 0.012), and T stage (HR: 1.253, 95% CI: 1.161–1.351, *p* < 0.001) (Table 2).

**Table 1:** Univariate Cox regression analysis of clinical and laboratory features associated with RFS in ccRCC patients.

Feature	HR	Lower 95% CI	Upper 95% CI	*p* value
Gender	1.334	1.002	1.774	0.048
Age	1.028	1.015	1.040	<0.001
Pathologic tumor size (cm)	1.340	1.295	1.387	<0.001
Nuclear grade	2.199	1.834	2.636	<0.001
Hemorrhage	1.162	0.798	1.694	0.433
Necrosis	2.520	1.762	3.605	<0.001
Nerve invasion	7.550	3.107	18.343	<0.001
Lymphovascular invasion	2.456	1.551	3.891	<0.001
T stage	1.804	1.664	1.955	<0.001
N stage	0.384	0.126	1.171	0.092
Neutrophil count	1.085	1.032	1.141	0.001
Monocyte	1.161	1.018	1.326	0.027
Lymphocyte count	0.642	0.507	0.812	<0.001
Platelet count	1.004	1.002	<0.001	<0.001
Hemoglobin	0.983	0.978	0.989	<0.001
Lactate dehydrogenase	1.002	1.001	1.003	<0.001
Albumin	0.900	0.874	0.927	<0.001
Serum calcium	1.014	1.003	1.024	0.013
Fibrinogen	1.006	0.996	1.016	0.238

**Table 2:** Multivariate Cox regression analysis of clinical and laboratory features associated with RFS in ccRCC patients.

Feature	HR	Lower 95% CI	Upper 95% CI	*p* value
Gender	1.152	0.916	1.451	0.227
Age	1.014	1.004	1.024	0.006
Pathologic tumor size (cm)	1.147	1.098	1.199	<0.001
Nuclear grade	1.313	1.126	1.531	0.001
Necrosis	1.156	0.834	1.601	0.384
Nerve invasion	3.343	1.308	8.548	0.012
Lymphovascular invasion	0.922	0.601	1.415	0.711
T stage	1.253	1.161	1.351	<0.001
Neutrophil count	1.015	0.961	1.071	0.592
Monocytes	1.087	0.916	1.289	0.339
Lymphocyte count	0.876	0.737	1.042	0.134
Platelet count	1.001	1.000	1.003	0.053
Hemoglobin	0.999	0.993	1.004	0.682
Lactate dehydrogenase	1.001	1.000	1.001	0.182
Albumin	0.978	0.953	1.004	0.101
Serum calcium	1.008	0.997	1.018	0.151

**Fig. 1:**
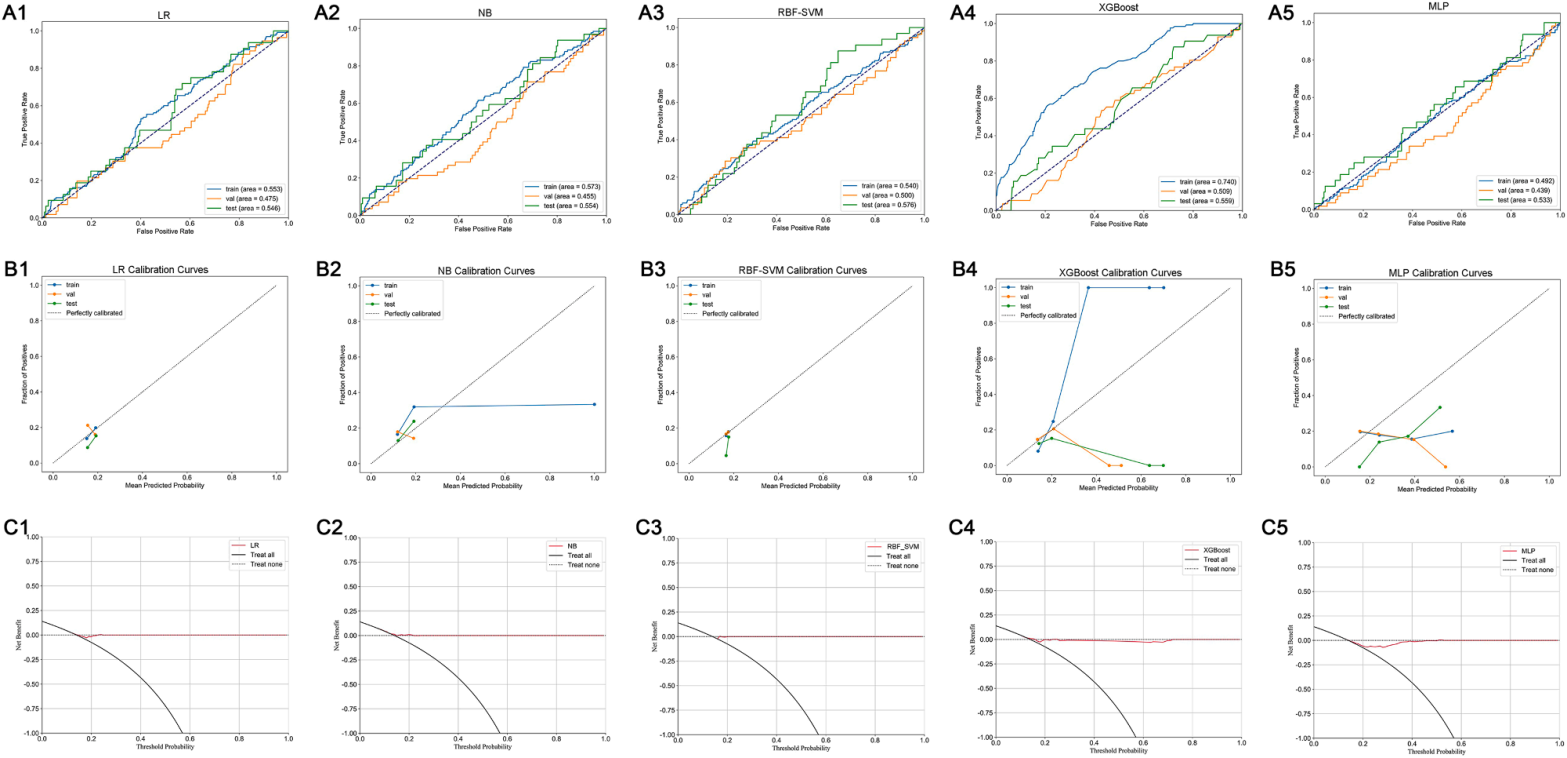
Comprehensive performance evaluation of ML models based on clinical features for RFS in ccRCC. (A1-A5) ROC curves show the discrimination ability of each model (LR, NB, RBF-SVM, XGBoost, MLP) in three cohorts including the training, validation, and external test cohorts. The diagonal dashed line represents the random probability (AUC=0.5). (B1-B5) Calibration curves are used to assess the consistency between predicted probabilities and observed outcomes, with the diagonal dashed line indicating perfect calibration. (C1-C5) DCA quantifies clinical net benefit and applicability relative to standard treatment strategies.

ML models built exclusively on clinical features showed unsatisfactory predictive capability for RFS in patients with ccRCC. Fourteen algorithms were assessed, with training AUC values ranging from 0.457 to 0.833, validation AUC from 0.439 to 0.562, and external test AUC from 0.419 to 0.576 (Supplemental Table 1). This narrow range of performance metrics further confirmed that clinical features alone lack sufficient discriminatory power. Among these models, RBF-SVM achieved the best performance, with an external test AUC of 0.576 (95% CI: 0.472–0.672), accuracy of 0.424 (95% CI: 0.365–0.485), sensitivity of 0.875 (95% CI: 0.719–0.969), specificity of 0.350 (95% CI: 0.279–0.424), and a validation AUC of 0.500 (95% CI: 0.412–0.572) (Fig. 1A3, Supplemental Table 1). Other competitive models, including LR (Fig. 1A1), NB (Fig. 1A2), XGBoost (Fig. 1A4), and MLP (Fig. 1A5), yielded inferior results. Most models also showed poor calibration in the external test cohort, with predicted probabilities inconsistent with actual recurrence rates, limiting their reliability for individual risk assessment. The calibration curves (Figs. 1B1-1B5) and decision curve analyses (Figs. 1C1-1C5) further revealed negligible net benefit compared to "treat-all" or "treat-none" strategies across most probability thresholds, indicating limited clinical utility when decisions rely solely on clinical features. Collectively, these findings demonstrate that clinical features alone are insufficient to accurately predict RFS in ccRCC, validating the necessity of the multimodal approach proposed in this study.

### Radiomics feature analysis and model performance

**Fig. 2:**
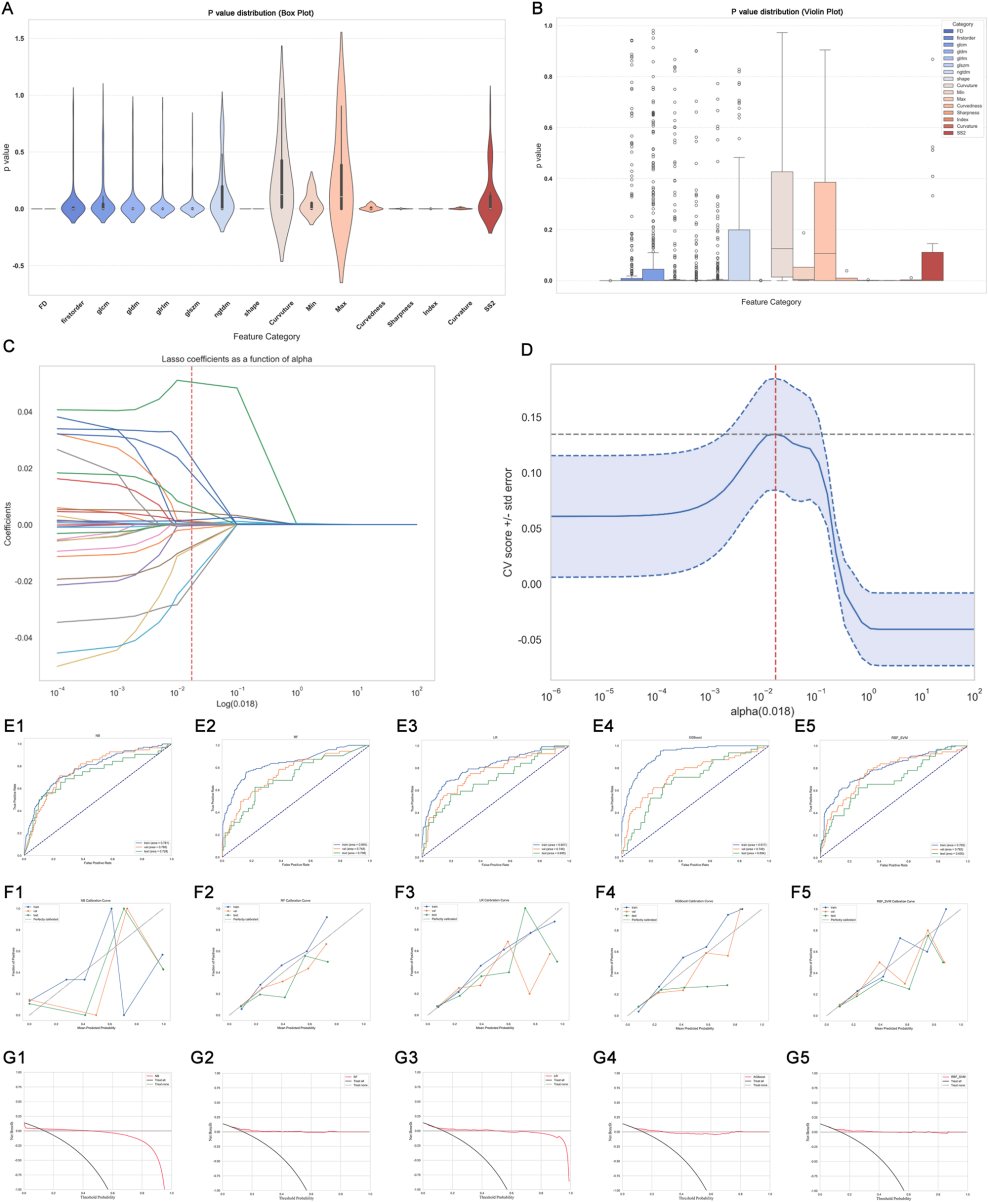
Radiomics workflow and model performance for RFS prediction in ccRCC. (A-B) Distribution of *p*-values for radiomics features across different categories. (C) LASSO regression coefficient profiles and the optimal α value determined by 10-fold cross-validation. (D) Performance curve of cross-validation. (E1-E5) ROC curves of each model in the three cohorts. (F1-F5) Calibration curves of each model in the external validation set. (G1-G5) DCA curves of each model.

A total of 1637 radiomics features were initially extracted from the segmented tumor volume. Univariate correlation analysis demonstrated that texture features possessed the strongest correlation with tumor recurrence (Fig. 2A and 2B). To eliminate redundant and low-predictive features, we performed stepwise feature selection including *t*-test, correlation analysis, and least absolute shrinkage and selection operator (LASSO) regression, and finally retained 31 features with non-zero coefficients. The retained features were dominated by texture features derived from multiple matrices, including 12 gray level co-occurrence matrix (GLCM) features, 8 gray level size zone matrix (GLSZM) features, and 6 gray level run length matrix (GLRLM) features, supplemented by 3 shape features and 2 fractal dimension measurement features. Correlation analysis confirmed that curvature-based topological features and multiple texture features [GLCM, GLRLM, neighborhood gray tone difference matrix (NGTDM)] were significantly correlated with RFS (all *p* < 0.05). The LASSO coefficient plot indicated that textural heterogeneity and complexity indicators carried the highest predictive weight for recurrence prediction (Fig. 2C). The LASSO coefficient path further visualized the dynamic feature selection process with the variation of regularization parameter α. Based on 10-fold cross-validation, the optimal α value was determined as 0.018, which screened out 13 stable core features from the high-dimensional feature space (Fig. 2D).

We subsequently constructed multiple ML models based on the screened radiomics features and systematically compared their predictive performance and generalization ability across training, validation, and external test cohorts (Fig. 2E1-2E5). Tree-based ensemble models showed prominent overfitting problems (Supplementary Table 2).

Among all included algorithms, the NB model exhibited the optimal generalization ability with the most stable performance and minimal overfitting. Its AUC values were 0.781 (95% CI: 0.738–0.826) in the training cohort, 0.768 (95% CI: 0.696–0.825) in the validation cohort, and 0.728 (95% CI: 0.606–0.820) in the external test cohort (Fig. 2E1). In the external test cohort, the NB model also achieved an accuracy of 0.834, a sensitivity of 0.531, and a specificity of 0.883, with robust predictive efficacy across all cohorts. Other mainstream models showed different degrees of performance attenuation in external validation. The RF model had AUC values of 0.840 (95% CI: 0.800–0.882), 0.748 (95% CI: 0.672–0.818), and 0.708 (95% CI: 0.604–0.806) in the three cohorts (Fig. 2E2). LR showed stable but mediocre performance, with corresponding AUC values of 0.807 (95% CI: 0.765–0.852), 0.746 (95% CI: 0.675–0.812), and 0.695 (95% CI: 0.570–0.785) (Fig. 2E3). The XGBoost model presented obvious overfitting, with a high training AUC of 0.917 (95% CI: 0.893–0.938) and a validation AUC of 0.748 (95% CI: 0.672–0.819), but a low external test AUC of 0.694 (95% CI: 0.563–0.788) (Fig. 2E4). For SVM models with different kernel functions, the RBF-SVM model obtained AUC values of 0.793 (95% CI: 0.750–0.838), 0.762 (95% CI: 0.685–0.830), and 0.690 (95% CI: 0.573–0.778) in the three cohorts, realizing balanced performance and no obvious overfitting (Fig. 2E5).

Calibration curve analysis (Fig. 2F1-2F5) revealed that despite the acceptable discriminative ability of all ML models, none achieved satisfactory probability calibration in the external test cohort, indicating that model recalibration is indispensable before clinical application. The DCA of all models (Figs. 2G1-2G5) further verified the clinical application value of all radiomic models. All models yielded favorable net benefit within the threshold probability range of 0%−35%, with the optimal clinical net benefit observed at 5%−15%, which supported their feasibility for postoperative risk stratification and individualized therapeutic decision-making for ccRCC. Notably, the established radiomic models significantly outperformed the models constructed solely with clinical data, fully validating the independent predictive value of quantitative CT-derived imaging features for ccRCC recurrence.

### DL model comparison and performance

The performance of four DL architectures for DL label generation was systematically evaluated across training, validation, and external test cohorts (Table 3). Overall, the 3D-ViT model outperformed all comparative models with the best comprehensive and robust predictive performance, followed by ResNet-18 and ResNet-50, while ResNet-101 exhibited severe overfitting and poor generalization ability.

**Table 3:** Performance metrics of DL models for RFS prediction in training, validation, and external test cohorts.

Model	Cohort	AUC	Accuracy	Sensitivity	Specificity	PPV	NPV	F1-Score
3D-ViT	Training	0.866 (0.835–0.894)	0.752 (0.721–0.782)	0.769 (0.692–0.838)	0.748 (0.714–0.780)	0.395 (0.358–0.435)	0.938 (0.921–0.956)	0.522 (0.478–0.568)
	Validation	0.852 (0.796–0.900)	0.738 (0.688–0.789)	0.768 (0.661–0.875)	0.732 (0.678–0.785)	0.381 (0.322–0.447)	0.936 (0.908–0.965)	0.509 (0.437–0.580)
	External test	0.846 (0.767–0.916)	0.769 (0.712–0.817)	0.750 (0.594–0.875)	0.772 (0.711–0.827)	0.348 (0.279–0.422)	0.950 (0.922–0.975)	0.475 (0.389–0.563)
ResNet-18	Training	0.866 (0.835–0.894)	0.752 (0.721–0.782)	0.769 (0.692–0.838)	0.748 (0.714–0.780)	0.395 (0.358–0.435)	0.938 (0.921–0.956)	0.522 (0.478–0.568)
	Validation	0.725 (0.658–0.793)	0.653 (0.606–0.703)	0.661 (0.536–0.768)	0.651 (0.590–0.709)	0.289 (0.243–0.344)	0.899 (0.868–0.932)	0.402 (0.338–0.470)
	External test	0.727 (0.626–0.815)	0.664 (0.603–0.725)	0.688 (0.531–0.844)	0.660 (0.594–0.721)	0.247 (0.191–0.306)	0.929 (0.894–0.962)	0.364 (0.281–0.441)
ResNet-50	Training	0.825 (0.786–0.859)	0.722 (0.688–0.753)	0.738 (0.662–0.808)	0.719 (0.684–0.753)	0.360 (0.320–0.398)	0.928 (0.907–0.945)	0.484 (0.433–0.528)
	Validation	0.753 (0.686–0.811)	0.675 (0.625–0.726)	0.714 (0.589–0.821)	0.667 (0.609–0.728)	0.315 (0.265–0.368)	0.916 (0.884–0.944)	0.437 (0.373–0.500)
	External test	0.703 (0.605–0.799)	0.668 (0.607–0.729)	0.656 (0.500–0.813)	0.67 (0.604–0.736)	0.244 (0.189–0.308)	0.923 (0.890–0.955)	0.356 (0.278–0.441)
ResNet-101	Training	0.889 (0.861–0.915)	0.795 (0.766–0.824)	0.769 (0.700–0.838)	0.801 (0.768–0.832)	0.452 (0.408–0.500)	0.942 (0.925–0.958)	0.570 (0.521–0.618)
	Validation	0.704 (0.632–0.777)	0.650 (0.599–0.700)	0.625 (0.500–0.750)	0.655 (0.598–0.713)	0.280 (0.230–0.336)	0.891 (0.858–0.924)	0.387 (0.317–0.459)
	External test	0.650 (0.552–0.748)	0.611 (0.541–0.677)	0.625 (0.469–0.781)	0.609 (0.538–0.675)	0.206 (0.152–0.263)	0.909 (0.870–0.947)	0.310 (0.230–0.390)

**Fig. 3:**
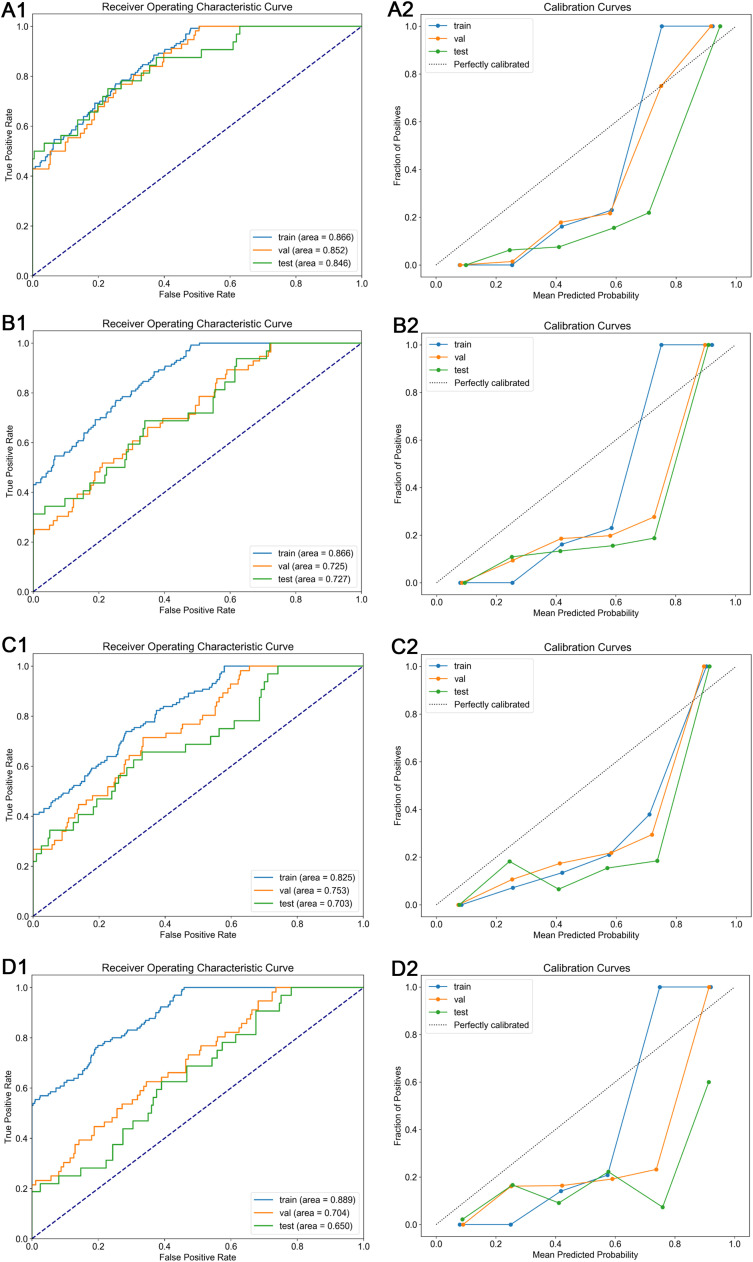
Performance comparison of different DL models for RFS prediction in ccRCC. (A1-A2) ViT model. (B1-B2) ResNet-18 model. (C1-C2) ResNet-50 model. (D1-D2) ResNet-101 model. The left panels show the ROC curves of each model in the training, validation, and external test cohorts, and the right panels present the model calibration curves.

Specifically, the 3D-ViT model achieved stable and high discriminative ability in all cohorts. It obtained an AUC of 0.866 (95% CI: 0.835–0.894) in the training cohort, 0.852 (95% CI: 0.796–0.900) in the validation cohort, and 0.846 (95% CI: 0.767–0.916) in the external test cohort (Fig. 3A1). For external test cohort quantitative indicators, the model yielded an accuracy of 0.769 (95% CI: 0.712–0.817), a sensitivity of 0.750 (95% CI: 0.594–0.875), and a specificity of 0.772 (95% CI: 0.711–0.827). The calibration curve further verified that the predicted probabilities of 3D-ViT were highly consistent with actual clinical outcomes (Fig. 3A2). The model presented favorable learning efficiency without overfitting, demonstrating excellent predictive stability and calibration performance.

Among the ResNet series models, ResNet-18 achieved the optimal comprehensive performance. It maintained consistent discriminative power across different cohorts, with AUC values of 0.866 (95% CI: 0.835–0.894) in the training cohort, 0.725 (95% CI: 0.658–0.793) in the validation cohort, and 0.727 (95% CI: 0.626–0.815) in the external test cohort (Fig. 3B1). Additionally, ResNet-18 showed good fitting status with reliable calibration curves in all cohorts (Fig. 3B2). ResNet-50 possessed balanced prognostic discriminative capability, with cohort AUCs of 0.825 (95% CI: 0.786–0.859, training), 0.753 (95% CI: 0.686–0.811, validation), and 0.703 (95% CI: 0.605–0.799, external test), accompanied by moderate overall model calibration (Fig. 3C1 and 3C2). In contrast, ResNet-101 showed obvious overfitting and poor generalization performance. Although it achieved the highest training cohort AUC of 0.889 (95% CI: 0.861–0.915) among all included models, its predictive ability dropped sharply in validation and external test cohorts, with AUCs of only 0.704 (95% CI: 0.632–0.777) and 0.650 (95% CI: 0.552–0.748), respectively (Fig. 3D1). The calibration curve of ResNet-101 further confirmed the presence of severe overfitting (Fig. 3D2), limiting its clinical application value.

**Fig. 4:**
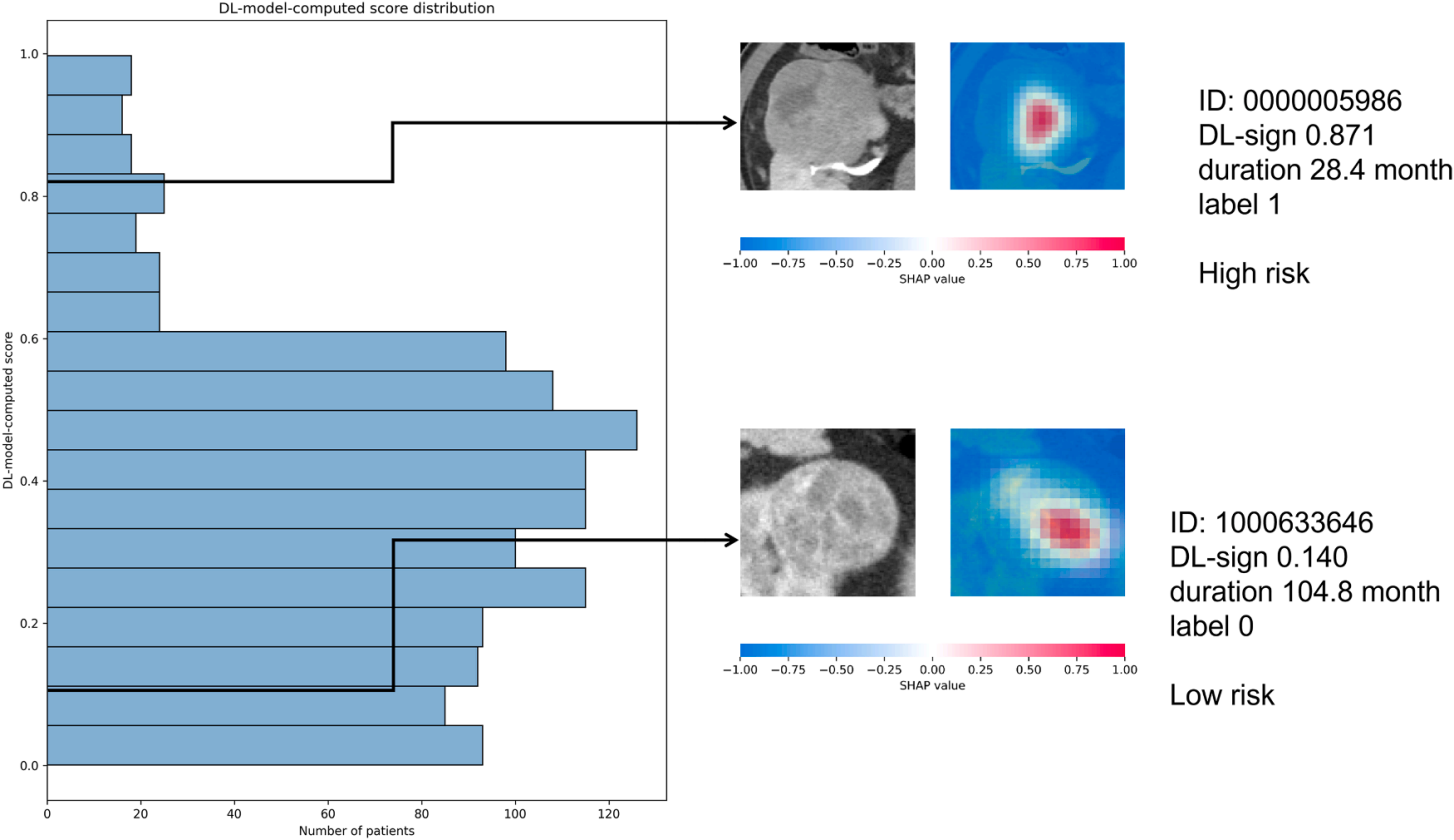
Decision mechanism interpretation of the DL model based on SHAP explainable analysis and typical clinical cases. The left panel displays the distribution of DL-sign scores for all enrolled patients, with representative high- and low-risk cases annotated. The upper and lower panels show CT images and SHAP heatmap visualization results of typical high-risk and low-risk cases, respectively.

To further interpret the decision-making mechanism of the optimal 3D-ViT model, SHAP interpretability analysis combined with typical clinical cases was performed (Fig. 4). The distribution of DL risk scores for all enrolled patients and representative high- and low-risk cases predicted by the model are displayed in Fig. 4. SHAP values ranged from −1.00 to +1.00, where negative values corresponded to low-risk prediction and positive values corresponded to high-risk prediction. The high-risk case (ID: 0000,005,986) had a DL-sign score of 0.871, and the patient suffered early tumor recurrence at 28.4 months after surgery. The superposition analysis of tumor CT images and SHAP heatmaps indicated that the red highlighted areas represented the key tumor features captured by the 3D-ViT model, which were the core basis for its high-risk prediction. For the low-risk case (ID: 1000,633,646), the DL-sign score was 0.140, and the patient remained recurrence-free during the 104.8-month follow-up period. Its unique SHAP feature attention pattern demonstrated that the model could accurately identify imaging phenotypes associated with favorable tumor prognosis. These results intuitively verified that the 3D-ViT model could precisely focus on clinically prognostic tumor regions of interest (ROI) and achieve explainable risk prediction.

The superior predictive performance and generalization ability of the 3D-ViT model are mainly attributed to its unique self-attention mechanism. This mechanism enables the model to effectively capture long-range dependency features and global contextual information of the entire tumor region, while SHAP-based visualization results further validate the model's targeted recognition ability for critical tumor functional regions, laying a solid foundation for its accurate and stable prognostic prediction.

### Integrated nomogram performance

**Fig. 5:**
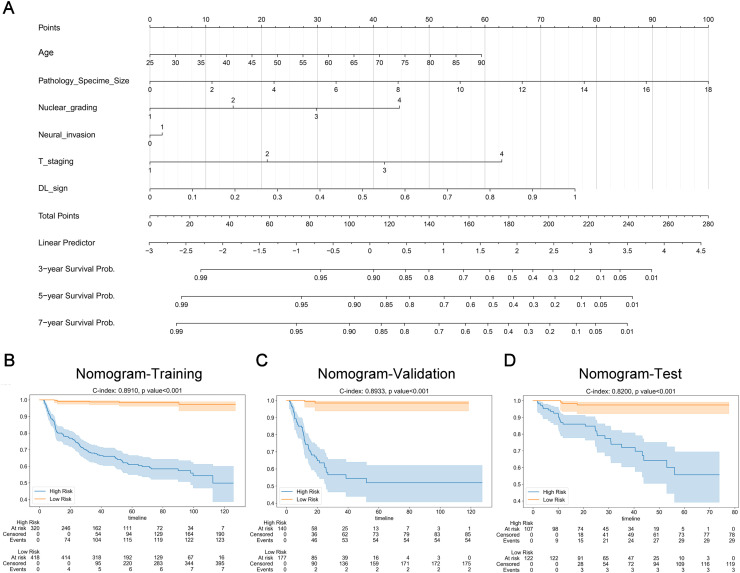
Risk stratification and integrated nomogram for RFS prediction. (A) Integrated nomogram combining clinical features and DL-sign for predicting 3-, 5-, and 7-year RFS. Total risk score was summed from the points of each variable, and the corresponding survival probability was read from the scale. (B-D) KM RFS curves for three cohorts grouped by the integrated nomogram. Patients were divided into low- and high-risk groups using the optimal cut-off value, with shaded regions indicating 95% CIs.

The integrated nomogram established in this study possessed superior predictive performance compared with single-modality prognostic models (Table 4). Notably, the Rad-sign was not included in the final model due to its consistently low C-index across all cohorts, limited incremental prognostic value, and prominent overfitting tendency in the external test cohort. This integrated model incorporated five conventional clinical features, including age, tumor size, nuclear grade, perineural invasion, and T stage, as well as a DL-sign (0–1) extracted from preoperative CT images via the 3D-ViT model (Fig. 5A). The Cox proportional hazards regression-based integrated nomogram achieved accurate patient risk stratification and robust prognostic discrimination across all independent cohorts. The concordance index (C-index) was 0.891 in the training cohort, 0.893 in the validation cohort, and 0.820 in the external test cohort (Fig. 5B-5D). Correspondingly, KM survival analysis confirmed that the model could significantly stratify patients into high- and low-risk subgroups with distinct RFS outcomes in all cohorts (all *p* < 0.001).

**Table 4:** C-index values of four prognostic models in the training, validation, and external test cohorts.

Model	Training (n = 738)	Validation (n = 317)	External Test (n = 229)
Integrated Nomogram	0.891	0.893	0.820
Clinical Feature	0.284	0.495	0.425
Rad-sign	0.188	0.271	0.320
DL-sign	0.485	0.481	0.592

**Fig. 6:**
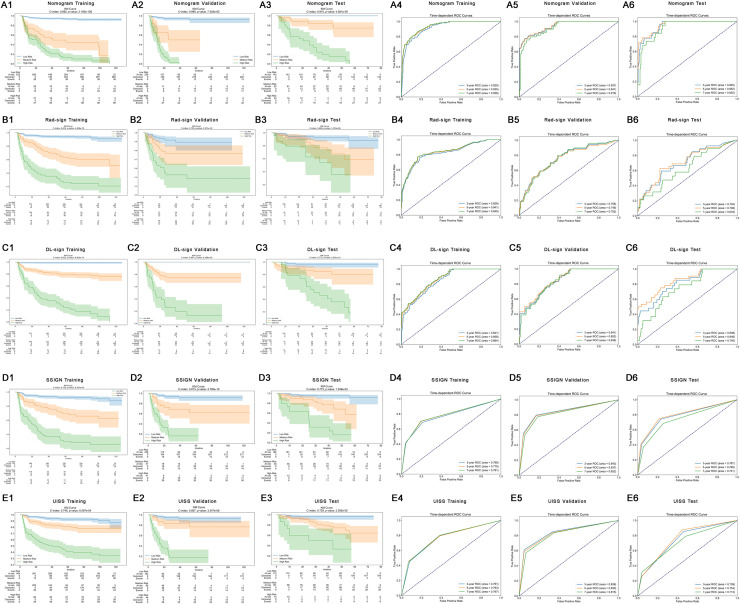
KM survival analysis and time-dependent ROC curves of the three prediction models for RFS, including the integrated nomogram model (A1-A6), Rad-sign (B1-B6), DL-sign model (C1-C6), SSIGN model (D1-D6), and UISS model (E1-E6). Each panel displays KM curves for low-, medium- and high-risk groups (blue, orange and green, respectively) as well as time-dependent ROC curves in the training, validation and external test cohorts. C-index and p values are provided for survival analyses, while AUC values at 3-, 5- and 7-year follow-up are marked on the time-dependent ROC curves to reflect model discrimination.

To enable systematic comparison across all prognostic models under a standardized framework, each model — including the integrated nomogram, Rad-sign, DL-sign, SSIGN, and UISS — was independently re-stratified into three risk groups (low, medium, and high risk) using two optimally determined cut-offs. Unlike the continuous-score C-index in Fig. 5, which is computed from the full spectrum of predicted risk values, the C-indices annotated on the KM plots in Fig. 6 are derived from these discrete group assignments and therefore measure how well the three-category stratification separates patients with different survival outcomes. KM survival curves for this three-group stratification are presented across training, validation, and external test cohorts in Fig. 6. Under this framework, the integrated nomogram effectively stratified patients into three distinct risk subgroups with significantly different RFS outcomes across all cohorts (Fig. 6A1-6A3). Time-dependent ROC analysis further demonstrated that the integrated nomogram maintained excellent discriminative ability at all follow-up time points, with 3-, 5-, and 7-year AUC values of 0.920, 0.935, and 0.938 in the training cohort (Fig. 6A4), 0.937, 0.941, and 0.919 in the validation cohort (Fig. 6A5), and 0.945, 0.952, and 0.920 in the external test cohort (Fig. 6A6), respectively.

Furthermore, the integrated nomogram outperformed the conventional UISS and SSIGN scoring systems across all cohorts. The Rad-sign risk stratification and time-dependent ROC curves are presented in Fig. 6B1-6B6, with 3-, 5-, and 7-year AUCs of 0.829, 0.841, and 0.845 in the training cohort (Fig. 6B4), 0.758, 0.748, and 0.752 in the validation cohort (Fig. 6B5), and 0.704, 0.708, and 0.633 in the external test cohort (Fig. 6B6). The DL-sign risk stratification and time-dependent ROC curves are presented in Figs. 6C1-6C6, with 3-, 5-, and 7-year AUCs of 0.841, 0.858, and 0.864 in the training cohort (Fig. 6C4), 0.841, 0.852, and 0.836 in the validation cohort (Fig. 6C5), and 0.808, 0.846, and 0.740 in the external test cohort (Fig. 6C6). The SSIGN system achieved 3-, 5-, and 7-year AUCs of 0.768, 0.779, and 0.781 in the training cohort (Fig. 6D4), 0.843, 0.837, and 0.822 in the validation cohort (Fig. 6D5), and 0.787, 0.799, and 0.741 in the external test cohort (Fig. 6D6), with corresponding KM risk stratification shown in Fig. 6D1-6D3. The UISS system yielded 3-, 5-, and 7-year AUCs of 0.761, 0.763, and 0.767 in the training cohort (Fig. 6E4), 0.839, 0.830, and 0.815 in the validation cohort (Fig. 6E5), and 0.739, 0.754, and 0.713 in the external test cohort (Fig. 6E6), with KM risk stratification shown in Fig. 6E1-6E3. Across all cohorts and time points, the integrated nomogram consistently demonstrated superior discriminative performance compared with the DL-sign alone, Rad-sign, SSIGN, and UISS scoring systems.

## Discussion

This multi-center study developed and validated an integrated ML framework that combines clinical features and DL-sign to predict RFS in patients with ccRCC following surgical resection. The principal findings are as follows: (1) clinical features alone demonstrated limited predictive capability (external test AUC: 0.576); (2) DL using a 3D-ViT captured additional prognostic information (external test AUC: 0.846); and (3) the integration of all two modalities achieved excellent discrimination (C-index: 0.910 in external test). These results significantly surpassed established prognostic systems (UISS C-index: 0.746, SSIGN C-index: 0.773).

The suboptimal performance of the models relying exclusively on clinical features was consistent with earlier studies, which reported C-indices around 0.82 [9]. While clinical parameters such as T stage, nuclear grade, and tumor size are established prognostic factors, they primarily capture macroscopic tumor characteristics and fail to reflect underlying biological heterogeneity. Incorporating laboratory biomarkers, including neutrophil count, lactate dehydrogenase, and albumin, offered incremental value in multivariate Cox analysis but remained insufficient for accurate individualized prediction within ML models.

Radiomics analysis demonstrated that quantitative imaging features can capture tumor heterogeneity patterns not discernible through conventional imaging interpretation. The 31 selected features were primarily texture metrics derived from GLCM, GLSZM, and GLRLM matrices, which reflect intra-tumoral heterogeneity in density, spatial organization, and contrast enhancement. These features are likely associated with pathological characteristics such as necrosis, hemorrhage, and hypoxia, which are established biological markers of aggressive tumor behavior [12,22]. The addition of fractal dimension and topological features based on curvature properties provided further insights into tumor complexity and spatial organization, potentially indicating the chaotic growth patterns typical of aggressive malignancies. However, despite the biological plausibility of these radiomic features, the corresponding Rad-sign exhibited consistently low predictive performance across all cohorts, with a C-index of only 0.188 in the training cohort, 0.271 in the validation cohort, and 0.320 in the external test cohort, significantly inferior to the DL-sign and clinical feature-based models. Moreover, the radiomic models demonstrated prominent overfitting tendencies and unsatisfactory probability calibration in the external test cohort, limiting their generalizability and clinical utility. Consequently, the Rad-sign was not incorporated into the final integrated nomogram, as it failed to provide incremental prognostic value beyond the combination of clinical features and the DL-sign.

The selection of a ML algorithm had a substantial impact on radiomics model performance. Among all included algorithms, NB exhibited the strongest generalization ability, achieving an external test AUC of 0.728, which is likely attributable to its unique probabilistic framework and inherent resistance to overfitting. This result highlights a persistent core challenge in radiomics research: the high dimensionality of radiomics features relative to the limited sample size greatly increases the susceptibility of ML models to overfitting during model construction and training [23]. In this study, we systematically compared the predictive performance of fourteen ML algorithms based on screened radiomics features. The comprehensive evaluation of model performance across different algorithms provides valuable methodological guidance for subsequent radiomics model development. Moreover, our findings further underscore the necessity of prioritizing external validation performance rather than relying solely on internal training and validation metrics when evaluating the robustness and clinical applicability of radiomics models.

The DL-sign based on 3D ViT successfully stratified patients into distinct risk groups, with time-dependent ROC curves showing sustained discrimination. The performance of 3D-ViT was notably superior compared to ResNet architectures. While ResNet-18, ResNet-50, and ResNet-101 achieved external test AUCs of 0.727, 0.703, and 0.650, respectively, the 3D-ViT achieved an AUC of 0.846, highlighting the significant advantage of self-attention mechanisms in medical imaging analysis. Unlike convolutional neural networks, which process information through local receptive fields, transformers capture long-range dependencies across the entire tumor volume via self-attention mechanisms [14,15], as visualized through SHAP analysis. This capability is particularly valuable for ccRCC, where prognostic features may encompass both local characteristics (such as texture and boundaries) and global patterns (including overall heterogeneity and spatial organization). The consistent performance across training (AUC: 0.866), validation (AUC: 0.852), and external test (AUC: 0.846) cohorts suggests effective learning without overfitting.

The integrated model's outstanding performance underscores the complementary value of the two feature modalities. The external test C-index of 0.910 and time-dependent AUCs of 0.945 at 3 years, 0.952 at 5 years, and 0.920 at 7 years indicate sustained discriminative ability over the follow-up period. Clinical features provide well-established prognostic information for staging and grading. DL features capture complex spatial patterns and contextual relationships. The integration of these modalities enables the model to leverage multiple independent sources of prognostic information, resulting in markedly improved discrimination compared to any single modality.

The superior performance over UISS and SSIGN scoring systems carries important clinical advantages. These established systems currently inform clinical decision-making regarding surveillance intensity and consideration of adjuvant therapy. In the external test cohort, UISS achieved a C-index of 0.746 with time-dependent AUCs of 0.739 at 3 years, 0.754 at 5 years, and 0.713 at 7 years. SSIGN showed comparable results, with a C-index of 0.773 and time-dependent AUCs of 0.787 at 3 years, 0.799 at 5 years, and 0.741 at 7 years. With a C-index improvement of 0.164 over UISS and 0.137 over SSIGN, the integrated model may help improve risk stratification. This advancement allows for identifying high-risk patients who might benefit from adjuvant therapy trials or increased surveillance, while reducing unnecessary interventions for low-risk patients. The model effectively stratified patients into three distinct risk groups with significantly different RFS outcomes (all *p* < 0.001), as demonstrated by the clear separation in KM curves.

Several limitations warrant consideration. First, although data were collected from multiple centers, all participating hospitals were located in China, potentially limiting generalizability to other geographic populations and healthcare systems. Consequently, validation in international cohorts is necessary. Second, the retrospective design introduces potential selection bias and issues with missing data, while conducting a prospective validation would provide stronger evidence. Third, while the model integrates comprehensive imaging and clinical features, it does not incorporate molecular or genomic data, which may offer additional prognostic value [24,25]. Future research could explore the integration of molecular biomarkers, such as gene expression signatures or somatic mutation profiles.

Fourth, the implementation of DL models requires significant computational resources and a high level of technical expertise. The 3D-ViT architecture requires substantial GPU memory to process volumetric CT data, which may restrict accessibility in resource-limited settings. The development of model compression techniques, knowledge distillation, or cloud-based deployment strategies could help address this limitation. Fifth, inter-observer variability in tumor segmentation, although minimized through standardized protocols, remains a potential source of measurement error that could impact radiomics feature reproducibility. Automated or semi-automated segmentation algorithms may enhance consistency [26].

Sixth, the study focused solely on the most common histological subtype of RCC, ccRCC. The model's applicability to other RCC subtypes, such as papillary or chromophobe, remains unvalidated and would require separate development and validation. Seventh, the model's performance in specific clinical scenarios, including distinguishing benign from malignant lesions or predicting response to particular therapies, was not assessed. Finally, the study did not evaluate the model's impact on clinical decision-making, patient outcomes, or cost-effectiveness. Implementation studies examining how the model influences surveillance strategies, treatment selection, and patient survival are necessary before widespread clinical adoption.

Despite these limitations, the study provides several important contributions. The systematic comparison of fourteen ML algorithms across three feature modalities offers comprehensive insights into optimal modeling strategies for ccRCC prognosis. Rigorous multi-center validation with independent external testing demonstrates generalizability beyond the training environment. Superior performance relative to established clinical scoring systems suggests potential for clinical translation. The integration of HCR, including advanced features such as fractal dimensions and curvature-based topological metrics, with DL-sign represents a comprehensive approach to extracting prognostic information from medical imaging.

Future research directions should include: (1) international multi-center validation to assess generalizability across diverse populations, imaging protocols, and healthcare systems; (2) integration of molecular and genomic biomarkers to capture biological heterogeneity beyond imaging features; (3) prospective validation studies with standardized imaging protocols and structured follow-up to confirm clinical utility; (4) development of interpretable models or explanation frameworks that provide transparent reasoning for predictions to facilitate clinical trust and adoption; (5) investigation of model performance in specific subgroups, such as partial versus radical nephrectomy, different T stages, and elderly patients, to identify potential limitations and opportunities for refinement; and (6) implementation studies evaluating the model's impact on clinical decision-making, patient management strategies, and health economic outcomes in real-world settings.

## Conclusion

This multi-center study validates that an integrated ML framework incorporating clinical features and 3D-ViT-derived DL features enables accurate and robust prediction of RFS in patients with ccRCC after surgical resection. In the independent external test cohort, the proposed integrated model yielded a C-index of 0.820 and maintained stable and powerful discriminative performance over longitudinal follow-up, with AUC values of 0.945 at 3 years, 0.952 at 5 years, and 0.920 at 7 years. Notably, this model significantly outperformed conventional prognostic scoring systems, including the UISS (C-index=0.729) and SSIGN (C-index=0.773) systems. Systematic evaluation of 14 ML algorithms across multiple feature modalities confirmed that algorithm selection is a critical determinant of model generalization performance. Specifically, the NB algorithm achieved the optimal predictive efficacy for radiomic feature analysis, and the 3D-ViT architecture exhibited superior performance over traditional conventional neural networks in DL-based risk stratification for ccRCC. Collectively, these findings highlight the clinical feasibility and value of multi-modal ML strategies for individualized postoperative risk assessment of ccRCC. This novel framework holds promising clinical prospects for optimizing surveillance intensity regimens and facilitating precise adjuvant therapy decision-making. Further prospective validation and international multi-center cohort studies are essential to verify the generalizability and clinical translational value of this model in diverse clinical practice settings.

## Institutional review board statement

This study was approved by the Ethics Committee of the Affiliated Tumor Hospital of Xinjiang Medical University in accordance with the Declaration of Helsinki.

## Informed consent statement

The requirement for written informed consent was waived due to its retrospective design and the anonymization of patient data.

## Funding statement

None.

## CRediT authorship contribution statement

**Yun-Lian Wang:** Writing – original draft, Formal analysis, Data curation, Conceptualization. **Feng Liu:** Formal analysis. **Qian-Biao Gu:** Conceptualization. **Qiang Liu:** Writing – original draft. **Ying Yang:** Writing – original draft. **Fang Cheng:** Supervision.

## Declaration of competing interest

The authors declare that they have no known competing financial interests or personal relationships that could have appeared to influence the work reported in this paper.

## Appendix Supplementary materials

Image, application 1

Image, application 2

**Supplementary Table 1.** Performance metrics of ML models built on clinical features. Image, application 3
