# Supplementary material for: Development and validation of an integrated machine learning model for recurrence-free survival prediction in clear cell renal cell carcinoma
Source: Transl Oncol. 2026 Jul 29;72:102937. doi: 10.1016/j.tranon.2026.102937 (PMC13449481; doi:10.1016/j.tranon.2026.102937)
Supplement: Supplementary file 1 [file mmc1.docx]

**Radiomics feature extraction and selection**

**Fractal dimension feature calculation**

The extraction process of fractal dimension features involved the following specific steps: First, all non-zero voxels were isolated to remove background noise and focus on the target region. A scale vector was constructed using a logarithmic scale, employing powers of 2 to enable multi-scale analysis, with the range determined by the maximum image size. A three-dimensional Box-Counting algorithm was used to estimate the voxel distribution at each scale: pseudo-randomly biased boxes were placed in the image space, and the number of voxels within each box was counted. This process was repeated multiple times to minimize random bias, and the results were averaged across scales. Subsequently, linear regression was performed on the relationship between voxel count and scale in a log-log coordinate system to identify the optimal fitting window, termed the fractal scale window. The selection of this window was based on the adjusted R^2^ coefficient to ensure sufficient linear correlation for the accurate estimation of the fractal dimension. The fractal dimension was calculated as the negative slope of the regression fit line. Statistical analysis of the calculated fractal dimensions included metrics such as mean, median, maximum, and minimum to provide a comprehensive understanding of the image's fractal characteristics.

**Curvature-based topological features**

Curvature properties describe the local geometric morphology of a surface, including the degree and directionality of bending. Specifically, Gaussian curvature reflects the overall bending nature and distinguishes between concave, convex, and flat regions. Mean curvature measures the local overall bending trend, while principal curvatures describe the extreme bending degree in specific directions. Higher-level geometric information, such as curvature magnitude and sharpness, was calculated to quantify bending intensity and sharpness. The shape index describes changes in geometric morphology through a continuous range of values, and total curvature measures the local overall absolute bending degree. Collectively, these properties provide a foundation for surface shape analysis, classification, and feature extraction, supporting complex geometric analyses and applications.

**Robust normalization**

Robust normalization was performed on the screened independent and stable HCR features, with the specific operation as follows: each feature column was subtracted by its median, and then divided by the interquartile range (IQR) to complete the normalization process.
